# Weather Information Seeking and Heat-Health Protective Actions During Pregnancy: An Exploratory Study

**DOI:** 10.3390/ijerph23070831

**Published:** 2026-06-24

**Authors:** Lisa K. Zottarelli, Robyn Stassen, Yejin Heo, Madeline Navarrete, Shamshad Khan, Thankam Sunil, Andrea Shields

**Affiliations:** 1 College of Social Work, University of Tennessee, Knoxville, TN 37996, USA; yheo1@vols.utk.edu; 2 Department of Public Health, College of Health, Community and Policy, University of Texas at San Antonio, San Antonio, TX 78249, USA; robyn.stassen@utsa.edu; 3 Department of Communication, College of Liberal and Fine Arts, University of Texas at San Antonio, San Antonio, TX 78249, USA; madeline.navarrete@my.utsa.edu (M.N.); shamshad.khan@utsa.edu (S.K.); 4 Department of Nutrition and Public Health Sciences, College of Education, Health, and Human Sciences, University of Tennessee, Knoxville, TN 37996, USA; tsunil@utk.edu; 5 Department of Obstetrics and Gynecology, University of Connecticut Health, Farmington, CT 06030, USA; ashields@uchc.edu

**Keywords:** extreme heat, pregnancy, information seeking, weather, behavioral adaptation, Protective Action Decision Model, risk communication, maternal health, environmental health, trimester, health protection, heat health

## Abstract

**Highlights:**

**Public health relevance—How does this work relate to a public health issue?**

- Pregnant individuals face elevated risks for adverse maternal and fetal health outcomes due to extreme heat.
- This study identifies the relationships between weather information seeking and heat-health protective actions.

**Public health significance—Why is this work of significance to public health?**

- Increased weather information seeking during extreme heat is associated with use of heat-health protective actions such as limiting time in the sun, staying hydrated, and spending time in air conditioning.
- Routine weather information seeking alone is not enough to prompt heat-health protective actions such as changing plans.

**Public health implications—What are the key implications or messages for practitioners, policy makers and/or researchers in public health?**

- Clinicians, meteorologists, and public health agencies should consider integrating extreme heat pregnancy guidance into patient counseling and public messaging.
- Leveraging moments of increased weather information seeking during extreme heat may support the adoption of protective health behaviors among pregnant individuals.

**Abstract:**

Extreme heat poses health risks during pregnancy, but little is known about how pregnant individuals seek weather information to engage in heat-health protective actions. This study examined associations between routine and event-driven weather information seeking and both routine physiological heat-health protective actions (i.e., limiting sun exposure, staying hydrated, and spending time in air conditioning) and higher-threshold adaptive behaviors (i.e., changing plans due to heat). A cross-sectional survey of 195 pregnant individuals in Bexar County, TX, USA, was conducted during the summer and fall of 2024. Descriptive and nonparametric analyses explored relationships across trimesters. Participants demonstrated high routine weather information seeking and greater weather information needs since becoming pregnant. Over half (51.3%) reported increased weather information seeking during excessive heat, with lower increases during the first trimester. During extreme heat, most respondents increased heat-health protective actions. Increased information needs during pregnancy were significantly related to heat-health protective actions. Routine weather checking showed weak or inverse relationships with changing plans, suggesting that routine weather awareness alone may not prompt changing plans. Trimester patterns indicated heightened information seeking and protective actions later in pregnancy. Findings highlight the importance of pregnancy-specific heat risk communication with trimester-specific guidance provided in clinical counseling, public health messaging, and meteorological communication.

## 1. Introduction

Extreme heat is linked to increases in morbidity [1] and mortality in the general population [2,3]. The health impacts of extreme heat are not evenly distributed [4,5]. Certain groups have heightened susceptibility to the heat due to physiological, social, and structural factors. Individual risk to extreme heat is increased during pregnancy [6,7], and little is known about how this population engages in heat risk mitigation.

Heat risk is a function of the heat hazard (i.e., high ambient temperatures), individual vulnerability, ability to engage in mitigation efforts to reduce exposure, and the resulting adverse health outcomes [8]. This susceptibility, in the absence of effective mitigation efforts, results in heat-related health harms. Being informed about weather conditions may be particularly important during high temperatures because heat often lacks a clear visual threat. Individuals must gather weather information from weather forecasts, alerts, and other mediated cues in order to recognize risk and respond effectively.

The Protective Action Decision Model (PADM) describes how people could use weather forecast information in a decision-making process to respond to environmental hazards such as extreme heat [9]. Yet, the PADM has rarely been applied to the context of extreme heat [10]. Applying components of the PADM, the purpose of this study is to explore relationships between routine and event-driven weather information seeking behaviors and the use of heat-health protective actions during pregnancy within the context of extreme heat. Understanding when pregnant individuals seek and respond to weather information may allow for a clearer understanding of the links between weather information and protective actions. This could support the development of tailored heat warning messages for individuals who are pregnant.

This study aligns with the United Nations Sustainable Development Goal 3, which is to ensure healthy lives and promote well-being for all at all ages [11]. It is especially relevant to indicator 3.1, which seeks to reduce global maternal mortality, and 3.2, which is directed to end preventable neonatal mortality, including mortality associated with preterm births and congenital defects. Additionally, this work supports the efforts of SDG 13 regarding taking action to combat the impacts of climate change [12], and specifically indicator 13.3, which seeks to improve education and awareness of human actions to improve adaptation and reduce the impact of climate change.

### 1.1. Pregnancy and Heat-Health Risks

Pregnant people are particularly vulnerable given the physiological demands of pregnancy, and emerging evidence links heat exposure to adverse maternal and fetal health outcomes [13]. Pregnant people are an often-overlooked at-risk population despite growing evidence that extreme heat and high ambient temperatures are associated with poor pregnancy outcomes [6]. In a scoping review of 84 research articles written in English that examined evidence of extreme heat and pregnancy outcomes, Syed et al. found preterm birth (i.e., <37 weeks of gestation), low birthweight, congenital anomalies, and stillbirth (i.e., fetal loss > 20 weeks of gestation) were the most common adverse outcomes [7]. Extreme heat exposure early in pregnancy was associated with neural tube defects, especially spina bifida [14]. High ambient temperatures and extreme heat were associated with preterm delivery [15]. Premature rupture of membranes was found to be associated with less intense heatwaves rather than the most extreme heat events [16]. Extreme heat exposure was associated with maternal hospitalizations for pregnancy complications [17] and severe maternal morbidity, such as cardiovascular conditions [16].

Pregnancies last approximately 40 weeks, and health risks associated with extreme heat exist throughout the pregnancy [8]. There is growing evidence of time-specific effects of extreme heat and high ambient temperature on maternal and fetal health outcomes [6,7,16,17]. For example, certain neural tube defects are believed to occur early in the first trimester of pregnancy [18], and extreme heat exposure during this time period has been found to be associated with an increase in neural tube defects. Exposure to extreme heat in the second trimester was associated with hospitalizations for pregnancy complications in both the second and third trimesters [17]. Additional days of extreme heat exposure in the first trimester were associated with pregnancy complications at childbirth, and an additional day of extreme heat exposure in the third trimester was associated with an increased likelihood of a hypertension diagnosis within the third trimester [7]. Congenital anomalies were associated with maternal heat exposure during the first trimester, preterm birth followed high ambient heat exposure during the second and third trimesters, and stillbirths were associated with heat exposure throughout the pregnancy [7].

### 1.2. Weather Information Seeking

Weather information seeking is a complex cue to action within risk communication [19], and it has not been well examined for extreme heat. Research on routine, day-to-day weather information seeking suggests that weather information habits are patterned by engagement and context [20]. Survey and qualitative studies of routine weather forecast use find that people interpret weather information through personal thresholds and shift to more deliberate information seeking when timing, safety, or material consequences become more salient [21,22]. During severe or extreme weather, people seek additional weather information from a variety of meteorological sources, including broadcast news, weather apps, and social media [23,24]. Within the context of extreme heat, Google searches conducted during National Weather Service heat alerts showed increases in information seeking about heat stroke/exhaustion, heat illnesses, and air conditioning [25].

### 1.3. Heat-Health Protective Actions

It is possible to mitigate or prevent heat-related health risks through specific individual actions such as staying hydrated, limiting time in the sun, spending time in air conditioning, or avoiding outdoor activities [26,27,28,29]. People, especially women, will engage in heat-health protective actions during heatwaves [28,29,30], but the use of heat-health protective actions has not been found to be consistent across location, type of protective action taken, or heat event [31,32]. Awareness of heat alerts does not always result in people taking protective actions [33,34].

During pregnancy, individual heat-health preparedness is complicated by physiologic changes and logistical and financial challenges experienced by the individual [35]. Often, the adoption of heat-health protective actions is recommended for “at-risk” populations, but people who are pregnant are less frequently identified as an at-risk population compared to other populations such as older adults and children [29,30,31,32,33,34,35,36]. Individuals must also have the ability to engage in behaviors that reduce heat exposure, and in some cases may need to alter or abandon planned activities. Examining the relationships between weather information seeking and heat-health protective actions provides insight into how risk awareness translates into both routine exposure mitigation and more disruptive forms of adaptation such as changing plans.

### 1.4. Protective Action Decision Model

The Protective Action Decision Model (PADM) describes how people engage in a decision-making process to respond to environmental hazards and disasters [9]. People engage in weather information seeking in routine weather and then shift their information seeking during periods of severe or extreme weather [23,24]. This episodic engagement with weather information, within the broader context of the ecosystem of risk communication, drives behavioral decisions to engage in protective actions [37]. While the PADM has been applied to a wide variety of hazards and disasters, these have often been hazards with strong visual cues such as hurricanes, and it has been less frequently applied to hazards with weak visual cues such as extreme heat [10].

**Figure 1:**
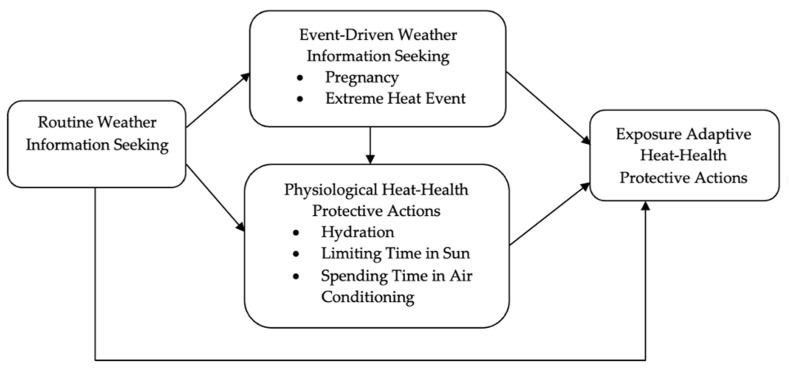
Theoretical Framing of Weather Information Seeking and Protective Heat-Health Actions.

In the present study, we utilize selected components of the PADM to examine the situational factors that lead to behavioral response. Within the context of extreme heat and pregnancy, as shown in Figure 1, we examine the routine and event-driven weather information seeking in relation to physiologically focused and adaptive heat-health protective actions. Event-driven weather information seeking considers perceived changes in information need while pregnant. It can also be examined as changes in weather information seeking within a threat-focused extreme heat context compared to the routine weather information seeking that is the individual’s perceived norm on a day-to-day basis. The heat-health protective actions are behavioral responses associated with the weather information. The first responses are actions that are physiologically focused to physically reduce risk by limiting time in the sun, staying hydrated, and spending time in air-conditioned environments. These actions are done within the context of existing daily activities and can be viewed as routine behavioral responses to high temperatures. The second type of protective heat-health action is exposure adaptation, in which individuals change plans to avoid exposure. Changing plans differs from the more routine actions focused on physiologically heat-health protective actions because changing plans requires moving beyond coping with heat to reorganizing life in response to heat. It is a risk avoidance strategy that cannot be done within existing daily activities. The categorization of two types of protective actions allows for differentiation between heat-health actions that occur within the context of routine activities and heat-exposure adaptation, which are meaningfully disruptive to daily life.

Based on theoretical framing, it was hypothesized that (H_1_) individuals who routinely engage in weather information seeking would be more likely to report increased event-driven weather information seeking while pregnant and during extreme heat events. (H_2_) Weather information seeking was expected to be associated with greater engagement with physiological heat-health protective actions. Likewise, (H_3_) weather information seeking was expected to be associated with a higher likelihood of having changed plans due to extreme heat. (H_4_) The physiological heat-health protective actions are expected to be associated with changing plans due to extreme heat. Finally, it is hypothesized that (H_5_) there will be increases in protective health actions in the final trimester of the pregnancy compared to the earlier trimesters.

## 2. Materials and Methods

The study data are from a cross-sectional survey of pregnant women who were 18 years of age or older and living in Bexar County, Texas, United States. The instrument was pretested in Spring 2025. Data collection was conducted from 22 May to 18 December 2024. San Antonio is the county seat of Bexar County and is the 7th most populous city in the United States. The geographic region experiences long hot summers and short mild winters. It averages 116 days per year at greater than 90 degrees Fahrenheit (F) (32.2 degrees Celsius (C)) [38].

### 2.1. Data Collection

Data were collected using a convenience sample with the participants recruited from childbirth classes, community pregnancy information fairs, health clinics, and social service agencies. Recruitment occurred primarily in person using a paper survey. An electronic version of the survey was available using Qualtrics, which was accessed through a QR code on flyers. The self-administered questionnaire was available in English and Spanish, although fewer than 5% took the survey in Spanish. As per the approved Institutional Review Board protocol and included in the informed consent statement, participants were given a $10 gift card upon completion of the survey. The survey took between 15 and 20 min to complete. This study was conducted in accordance with the Declaration of Helsinki and was approved by the Institutional Review Board of The University of Tennessee, Knoxville, TN, USA (UTK IRB-24-07988-XM). Informed consent was obtained from all subjects involved in the study.

### 2.2. Sample

Of the 202 people who consented to participate in the study and met the inclusion criteria of being 18 years of age or older, pregnant, and living in Bexar County, 195 completed the questions used in this analysis. As shown in Table 1, the sample was predominantly ethnically Hispanic (61.3%). Sixty-eight percent self-identified as white, 14.2% as Black, and 17.9% as another race or multiracial. About a third had a high school diploma. Forty-three percent had a household income of $30,000 or less and 19.4% had a household income greater than $75,000. One in ten participants indicated having a precarious housing situation. The majority of the participants were in their third trimester of pregnancy. While we have no way of comparing the sample to the actual pregnant population in San Antonio, TX, USA, the results were similar to the general population in terms of ethnicity (i.e., the City of San Antonio Hispanic population was 64.6%, had a bachelor’s degree or higher of 29.0%, and a median household income of $65,056) [39].

### 2.3. Measures

#### 2.3.1. Routine Weather Information Seeking

Routine weather information seeking refers to the frequency at which people check weather information or watch/listen to a weather forecast when conditions are within a normal range for the season and no weather watches, warnings, or advisories are active. To determine the routine weather information seeking behaviors, participants were asked, “In a normal week, how often do you check the weather information?” The response categories were coded every day (3), most days (2), some days (1), and rarely/never (0).

#### 2.3.2. Event-Driven Weather Information Seeking

Weather information seeking during pregnancy refers to a self-assessed change in routine weather information seeking that the participant has experienced since becoming pregnant. Respondents were given the statement, “Since I became pregnant, I need”, with response categories of “more information about the weather”, “less information about the weather”, and “about the same information about the weather”. Given the distribution of responses, with few respondents selecting needing less information, this variable was recoded into two categories. First, needing more information about the weather was coded as 1. The needing less or about the same information response categories were combined into a single category of not needing more information about the weather, and this was coded as 0.

Weather information seeking during excessive heat refers to changes in weather information seeking that occur when excessive heat is forecasted. “Excessive heat” is a term used by the National Weather Service to communicate to the public that outside ambient temperature conditions could or do present a threat to human health [40]. The City of San Antonio often uses the term “extreme” heat to reference outside ambient temperature conditions that could or do present a threat to human health, but they also direct the public to the National Weather Service for weather alerts [41]. Therefore, we used “excessive heat” because this is the term that people would be more likely to hear from broadcast meteorologists and from information they are directed to seek at the National Weather Service. The survey instructions explained that “excessive heat” referred to “abnormally hot summer weather” and individuals relied on their own understanding of excessive heat within this broader context. The respondents were asked, “If there was excessive heat in the weather forecast, would you check the local weather for updates?” The response categories were “I would not do this”, “less than I normally do”, “same as I normally do”, and “more than I normally do”. The responses were recoded into two categories. The first combined “I would not do this”, “less than I normally do”, and “same as I normally do” to create a “not more than normal” category and was coded as 0. “More than I normally do” was coded as 1.

#### 2.3.3. Physiological Heat-Health Protective Actions

Limiting time in the sun, staying hydrated, and spending time in air conditioning are heat-health protective actions that are physiologically focused and can be done within existing daily activities to mitigate risk. Three actions were included in this study. The first is limiting time in the sun. This is a heat-health protective action where one reduces time spent in the sun through spending time in the shade or going inside due to extreme heat. Respondents were asked, “If there was excessive heat in the weather forecast, would you limit your time in the sun?” The second physiological protective action was staying hydrated. To measure their actions toward staying hydrated, respondents were asked, “If there was excessive heat in the weather forecast, would you drink plenty of water to stay hydrated?” Spending time in air conditioning was the third physiological protective action to mitigate risks from extreme heat. For spending time in air conditioning, respondents were asked, “If there was excessive heat in the weather forecast, would you spend time in air conditioning?” To capture the reported changes in behaviors during extreme heat for each one of the three actions, the response categories were “more than I normally do”, “same as I normally do”, and “less than I normally do”. Similar to the event-driven weather information seeking variables, few respondents selected doing the action less than normal. Therefore, the responses of same and less than normally were combined into a “not more than normal” category and coded as 0. More than normal was 1.

#### 2.3.4. Exposure Adaptation of Changing Plans

The final heat-health protective action included in this study was the exposure adaptation of changing plans due to extreme heat. For this variable, respondents were asked to respond to the statement, “I change plans when there is excessive heat.” The response categories were always (3), often (2), sometimes (1), and rarely/never (0).

### 2.4. Analysis Plan

Descriptive statistics were run for all variables. Given that the variables are ordinal and that the purpose of the study was to examine associations rather than determinants between components included in the theoretical framework provided in Figure 1, chi-square ($\chi^{2})$ was used to test the relationships between variables, and Somers’ D (D) was used to report the direction and strength of those relationships. Finally, Kruskal–Wallis H was used to determine if there were relationships between trimesters for each of the variables. As a nonparametric test, it is generally robust to unequal group sizes if there are at least 3 groups with 5 or more cases in each group [42]. The analysis was conducted using IBM SPSS 31.0.0.0 (117) [43].

## 3. Results

### 3.1. Descriptive Statistics

Table 2 shows the frequencies and percentages for the variables for the whole sample of 195 respondents and by trimester. Of the pregnant women in the sample, 39.0% check the weather daily and 31.8% check the weather most days. This pattern of routine weather information seeking, either daily or most days, was consistent across the trimester subgroups, with between 70.0% and 72.2% of respondents in each trimester engaging in this action. Forty-five percent of respondents reported needing more weather information since becoming pregnant, and this was consistent across the three trimesters. When considering changes in weather information seeking during extreme heat, 51.3% reported checking the weather more frequently during extreme heat. In the first trimester, 35.7% reported increased extreme heat weather information seeking compared to 55.6% of respondents in their second trimester and 51.2% in their third trimester.

More than three-quarters of respondents reported increasing their use of physiological heat-health protective actions. For limiting time in the sun, 81.0% reported increasing this action. This action was reported more frequently among respondents in their first and third trimesters at 85.7% and 85.0%, respectively, compared to the second trimester (70.4%). Spending time in air conditioning had a similar pattern of greater frequency of reported increases in the first (85.7%) and third (81.1%) trimesters compared with the second trimester (70.4%). In total, 83.6% of respondents reported increasing their hydration during extreme heat, and the percentage increased from 78.6% in the first trimester to 79.6% in the second trimester and 85.8% in the third trimester.

For the exposure adaptation of changing plans, respondents reported having always (27.2%) or often (34.9%) changed plans during extreme heat. When examining this action by trimester, 57.2% of respondents in their first trimester, 68.5% of respondents in their second trimester, and 59.9% of respondents in their third trimester reported always or often changing plans due to extreme heat.

### 3.2. Bivariate Analyses Between Routine and Event-Driven Weather Information Seeking

It was hypothesized that individuals who routinely engage in weather information seeking would be more likely to report increased event-driven weather information seeking. Table 3 shows the results of the chi-square and Somers’ D analyses for this hypothesis for the whole sample and by trimester. Routine weather information seeking and event-driven weather information seeking during extreme heat had a weak negative association, meaning that routine weather information seeking was inversely related to event-driven extreme heat weather information seeking for the whole sample (D = −0.18) and for the subsample in their third trimester of pregnancy ($\chi^{2}$ = 8.89, *p* < 0.05; D = −0.12, *p* < 0.05). There were no statistically significant relationships between routine weather information seeking and increased need for weather information during pregnancy.

### 3.3. Bivariate Analyses Between Weather Information Seeking and Physiological Heat-Health Protective Actions

Weather information seeking, both routine and event-driven, was hypothesized to be associated with greater engagement with the physiological heat-health protective actions of limiting time in the sun, staying hydrated, and spending time in air conditioning. Table 4 shows the results of the bivariate analyses for the three weather information seeking variables and the physiological heat-health protective action of limiting time in the sun. There was no statistically significant relationship between routine weather information seeking and limiting time in the sun. Reporting a need for more weather information during pregnancy had a weak positive statistically significant relationship with limiting time in the sun ($\chi^{2}$ = 5.72, *p* < 0.05; D = 0.14) for the whole sample. For respondents in the second trimester, there was a statistically significant weak positive relationship between needing more weather information since becoming pregnant and limiting time in the sun ($\chi^{2}$ = 3.48, *p* < 0.10; D = 0.23, *p* < 0.05). The relationship between checking weather more frequently in extreme heat and limiting time in the sun was statistically significant during the whole pregnancy ($\chi^{2}$ = 16.08, *p* < 0.001; D = 0.23, *p* < 0.001) and among respondents in the second ($\chi^{2}$ = 8.60, *p* < 0.01; D = 0.37, *p* < 0.01) and third ($\chi^{2}$ = 8.12, *p* < 0.01; D = 0.18, *p* < 0.01) trimesters.

The analyses of the relationships between weather information seeking behaviors and the physiological heat-health protective action of staying hydrated are provided in Table 5. The chi-square between routine weather information seeking and staying hydrated was statistically significant ($\chi^{2}$ = 8.65, *p* < 0.05). The need for more weather information since becoming pregnant and staying hydrated was positive and statistically significant ($\chi^{2}$ = 4.21, *p* < 0.05; D = 0.11, *p* < 0.05) for the whole sample and in the second trimester ($\chi^{2}$ = 3.86, *p* < 0.05; D = 0.22, *p* < 0.05). The relationship between increasing weather information seeking during an extreme heat event and staying hydrated was statistically significant for the whole sample ($\chi^{2}$ = 4.38, *p* < 0.05; D = 0.11, *p* < 0.05) and during the second trimester ($\chi^{2}$ = 4.48, *p* < 0.05; D = 0.23, *p* < 0.05).

Table 6 shows the results of the analyses between weather information seeking and spending time in air conditioning. The Somers’ D showed a weak inverse statistically significant relationship between routine weather information seeking and spending time in air conditioning (D = −0.10, *p* < 0.05). Reporting a need for more weather information since becoming pregnant was statistically significant with spending time in air conditioning ($\chi^{2}$ = 5.57, *p* < 0.05; D = 0.20, *p* < 0.05). In the third trimester, there was a weak statistically significant positive relationship between weather information needs during pregnancy and spending time in air conditioning ($\chi^{2}$ = 2.95, *p* < 0.10; D = 0.12, *p* < 0.05). In the whole pregnancy, the relationship between weather information seeking during extreme heat and spending time in air conditioning was statistically significant ($\chi^{2}$ = 13.49, *p* < 0.001; D = 0.32, *p* < 0.001). This relationship was also statistically significant in the second ($\chi^{2}$ = 8.60, *p* < 0.01; D = 0.12, *p* < 0.01) and third ($\chi^{2}$ = 5.74, *p* < 0.05; D = 0.27, *p* < 0.05) trimesters.

### 3.4. Bivariate Analyses Between Weather Information Seeking, Physiological Heat-Health Protective Actions, and Exposure Adaptation of Changing Plans

Table 7 shows the results of the bivariate analyses between the three weather information seeking variables and changing plans, as well as the relations between the three physiological heat-health protective actions and changing plans due to extreme heat. For the whole sample, as well as during the second and third trimester, the relationships between routine weather information seeking and changing plans were statistically significant but weak and inversely related ($whole\;sample:\;\chi^{2}$ = 15.36, *p* < 0.10; D = −0.19, *p* < 0.05; second trimester: D = −0.29, *p* < 0.05; third trimester: D = −0.19, *p* < 0.05). The relationship between needing more weather information since becoming pregnant and changing plans was statistically significant ($\chi^{2}$ = 12.34, *p* < 0.001; D = 0.18, *p* < 0.05). Increased weather information seeking during extreme heat and changing plans were statistically significant during the whole pregnancy ($\chi^{2}$ = 12.84, *p* < 0.001, D = 0.24, *p* < 0.001) and in the third trimester ($\chi^{2}$ = 9.57, *p* < 0.05; D = 0.29, *p* < 0.05). For the physiological heat-health protective actions associated with changing plans, limiting time in the sun was statistically significant in the whole pregnancy ($\chi^{2}$ = 10.34, *p* < 0.05; D = 0.27, *p* < 0.05) and in the third trimester ($\chi^{2}$ = 18.16, *p* < 0.001; D = 0.50, *p* < 0.001). Additionally, staying hydrated was statistically significant during the whole pregnancy ($\chi^{2}$ = 11.21, *p* < 0.05; D = 0.22, *p* < 0.10) and in the third trimester ($\chi^{2}$ = 12.91, *p* < 0.01; D = 0.32, *p* < 0.05).

### 3.5. Comparison Across Trimesters

Finally, to examine the hypothesis that there would be statistically significant differences between trimesters for each of the variables, Kruskal–Wallis H tests were conducted. Only limiting time in the sun was statistically different by trimester (Kruskal–Wallis H = 5.49, df = 2, *p* = 0.064). The remaining variables did not show statistically significant differences between trimesters of pregnancy.

## 4. Discussion

This study contributed to understanding the relationships between weather information seeking and heat-health protective actions across pregnancy. It was guided by selected components of the PADM [9,44]. Participants reported frequent routine weather information seeking, with more than two-thirds checking the weather daily and another third checking the weather on most days. About half of the respondents said that they would check the weather more frequently when excessive heat was forecast. This supports the application of the PADM to extreme heat in the area of routine information engagement, shifting to episodic event-driven engagement in periods of risk salience [9].

Despite the high frequency of engagement with routine weather information, this behavior was often weakly and inversely related to heat-health protective actions. Conversely, extreme heat weather information seeking was often positively and more strongly associated with heat-health protective actions. The PADM suggests that routine engagement with information would likely be insufficient, as it does not necessarily move the predecision process of exposure to information towards threat perception and eventual action [35]. In the case of heat as a threat, with its lack of visual cues, as would be found with storms and other weather hazards, individuals may fail to recognize the need to evaluate heat risks. Routine weather information seeking may be habituated and serve a purpose that does not result in a health risk evaluation. Alternatively, summers can be long, and temperatures often rise over time and may stay high for an extended period. It is possible that high temperatures become normalized in the process of routine weather information seeking, potentially creating a gap in recognizing a heat threat and moving towards adopting heat-health protective actions. This disconnect between routine weather information seeking and heat-health protective actions suggests an important area in need of further examination to understand the cues that prompt the shift to event-driven weather information seeking actions within the context of extreme heat. Event-driven increases in weather information seeking were less common in the first trimester (35.7%) than in the second (55.6%) and third (51.3%), potentially suggesting that risk salience may change as pregnancy progresses.

Most respondents reported increasing the use of physiological heat-health protective actions, such as limiting time in the sun, staying hydrated, and spending time in air conditioning, but not the adaptive heat-health protective action of changing plans. Conceptually, the heat-health protective action typology used in this study provides guidance to differentiate heat-health protective actions based on the disruption to daily activity that the action requires of the individual. Limiting time in the sun, staying hydrated, and spending time in air conditioning require low-to-moderate response complexity and can be undertaken as part of daily activities. These can be understood as coping strategies to address physiological needs during extreme heat exposure. These actions are undertaken to protect oneself from the heat but are not disruptive. On the other hand, changing plans represents a disruptive adaptive response indicative of elevated perceived risk or constrained coping capacity within the context of routine activities. This typology aligns with the PADM’s pathway from cues and information to protective action decision-making, which anticipates changes in protective actions [9,44].

This study centers pregnant individuals in the examination of weather information seeking and heat-health protective actions. Pregnancy represents a complex vulnerability to disasters and extreme weather. Unlike other forms of health-related vulnerabilities, which tend to be either stable or progressive over time, pregnancy is a time-limited vulnerability. Pregnancy lasts less than 40 weeks. People who are pregnant may not realize that they are vulnerable to adverse health outcomes due to extreme heat. This study found an increasing willingness to engage in heat-health protective actions in the later trimesters. The relationship between weather information seeking in extreme heat and heat-health protective actions was inconsistent. Creating awareness about heat-health risks during pregnancy may require multiple sources, channels, and messengers. The American College of Obstetricians and Gynecologists recommends assessing environmental exposures, including extreme heat, and counseling patients accordingly [45]. Beyond healthcare settings, broadcast meteorologists and community social service providers could act as trusted intermediaries, explicitly naming pregnancy as an at-risk condition and prompting the use of heat-health protective actions [46]. This would embed pregnancy-specific heat-health guidance in extreme heat risk messaging.

### Limitations

This cross-sectional study used a convenience sample of pregnant individuals in Bexar County, TX, USA, and this limits the ability to establish causality, examine change over time, generalize, and geographically transfer the results of the study. As a cross-sectional study, causality cannot be determined because it is not possible to establish the temporal ordering of whether the information seeking preceded the heat-health protective actions or if some other confounding factor may have influenced both weather information seeking and heat-health protective actions. Further, while we compared individuals in different trimesters, we cannot determine if those differences are a result of changes over time or some other factor. Gathering a random sample of pregnant people in a geographic location was not possible, so we had to use a convenience sample. This presents a significant limitation to generalizability because people who attend prenatal care visits, childbirth classes, and other pregnancy-related services may not reflect the experiences of all pregnant women. Finally, this study has limited geographic transferability to other climates or care contexts. South Texas has hot summers and there is an expectation of being uncomfortably hot much of the summer. It has significant heat-mitigating infrastructure, including the widespread availability of air conditioning. These conditions may not be present in other locations.

A second limitation is the fact that the sample skewed heavily toward the third trimester of pregnancy, with few participants in the first trimester. Women often do not know they are pregnant until well into the first trimester. Prenatal care starts in the fourth week of pregnancy, with women attending prenatal check-ups once a month until week 28 and then increasing frequency of visits to twice a month and eventually once a week in the last month of pregnancy [47]. Childbirth classes are often taken mid-second trimester to early third trimester [48]. The clinics, childbirth classes, and social service agencies that provide pregnancy-related services where participants were recruited for this study were more likely to have patients and clients in the latter half of the pregnancy, thereby resulting in an imbalanced sample. Given that only five respondents were in their first trimester, and that five is the minimum for any group size using the Kruskal–Wallis H, the test may be underpowered. Future research would need to more actively recruit participants in the earlier weeks of pregnancy, and that could include quota sampling approaches to ensure more participants in the first and early second trimesters.

The measures were self-reported, and several constructs were recoded by dichotomizing multi-category items due to a small number of responses in one of the three categories. This recoding potentially attenuated associations and presents a potential threat to internal and external validity. This study was exploratory, and future research will require the refining of the measures.

Heat-related terminology presents challenges in research and communication to the public. In general, the public’s understanding of heat-related terminology is often incomplete and inconsistent [49]. “Excessive heat” reflected respondents’ interpretation of forecasts rather than standardized exposure metrics, a recognized limitation in pregnancy heat-health research that complicates comparability across studies [7]. The lack of a standardized or objective definition of “excessive heat” limits comparability across studies. Local governments, such as San Antonio, use both excessive heat and extreme heat to reference high temperatures and high heat indexes [41]. This is symptomatic of the National Weather Service’s reliance on jargon and technical language, which can create confusion among the public [49]. Further, the threat to human health posed by hot weather does not have a universal temperature and/or heat index threshold because the alert and advisory thresholds differ based on deviation from normal weather in a location [50].

Operationalizing “extreme heat” remains a methodological challenge, with multiple definitions and differing exposure metrics complicating the ability to compare and generalize research results as well as provide guidance to the public. The environmental thresholds identified for local heat action responses are often based on deviations from normal temperature, sometimes coupled with humidity, for specific locations. It is unclear if these location-specific environmental heat conditions align with heat risk awareness or understanding among vulnerable populations, including those who are pregnant.

Data collection occurred over a six-month period due to the complexities of reaching this population. This introduced a potential limitation because of the differing weather conditions that occurred throughout the data collection period. The daytime high temperatures varied between May and December. May had a maximum temperature of 90.8 F (32.7 C), followed by maximum daily temperatures of 95.8 F (35.4 C) in June, 94.3 F (34.6 C) in July, 99.2 F (37.3 C) in August, 93.3 F (34.1 C) in September, 90.6 F (32.6 C) in October, 79.8 F (26.0 C) in November, and 70.6 F (21.4 C) in December [51]. At the time, 2024 was the hottest year on record [52]. Given these conditions, there would have been media coverage about the heat. Therefore, the timing and environmental conditions during data collection could have influenced responses. Future research should examine the influence of current weather conditions on public perceptions of extreme heat and related heat health.

Finally, this study is limited because it was an exploratory study with a small sample size. The analyses relied exclusively on descriptive and bivariate nonparametric tests to examine the associations between components. Using multivariate models that could adjust for potential confounding factors such as age, income, education, and trimester was not conducted due to data limitations of sample size and distribution. Future research should include studies with larger sample sizes to allow for multivariate models to be conducted that include potential confounding sociodemographic, residential, environmental, and gestational factors.

## 5. Conclusions

This study seeks to examine how routine and event-driven weather information seeking is associated with heat-health protective actions during pregnancy. The results suggest that the relationships between these behaviors are complex. A general weather awareness through daily or near-daily weather information seeking does not appear sufficient to prompt heat-health protective actions, but extreme heat event-driven weather information seeking was associated with heat-health protective actions, especially those that could be incorporated into daily activities. The differences in relationships found between routine weather information seeking and extreme heat event-driven weather information seeking and the types of heat-health protective actions suggest that the two may operate differently in prompting individual efforts to reduce heat-health risks. Future researchers may want to explore the potential causal nature of these relationships and whether increasing weather awareness through event-driven weather information seeking is a key component to a stronger or more effective individual heat-health response.

This study was conducted in a location known for its hot summers. It has widespread availability of air conditioning that can be used as a reprieve from the heat. There are many cities with similar climates and built environment conditions. Additionally, Bexar County has a robust heat-health campaign every spring, providing an opportunity to build awareness about the vulnerability of pregnant individuals to extreme heat. Future research should continue to examine how pregnant individuals make heat-health decisions within the context of weather information, as well as other factors external to the individual, including the built environment and green spaces.

Pregnancy is a period of heat-health vulnerability, and the health implications change over the course of the pregnancy [6,7,14,15]. This study contributed to an understanding of how weather information seeking and the use of heat-health protective actions may differ across pregnancy trimesters. While the unequal sample sizes in each trimester require that the results be viewed cautiously, continued exploration of the potentially changing relationships between weather information seeking and use of heat-health protective actions at various timepoints in the pregnancy is warranted. This study underscores the need for pregnancy-specific heat-health communication that frames heat as a modifiable risk with clear action steps throughout the pregnancy.

## Figures and Tables

**Table 1:** Sample demographics.

Demographics		Number	Percent
Trimester			
	First	14	7.2%
	Second	54	27.7%
	Third	127	65.1%
Hispanic			
	Yes	119	61.3%
	No	75	38.7%
Race			
	White	133	68.2%
	Black	27	13.8%
	Other and Multiracial	35	17.9%
Education			
	Less than High School Diploma	9	4.6%
	High School Diploma	61	31.4%
	Some College/Associate’s Degree	68	35.1%
	Bachelor’s Degree	38	19.6%
	Graduate or Professional Degree	18	9.3%
Income			
	$30,000 or less	80	43.0%
	Between $30,000 and $45,000	32	17.2%
	Between $45,001 and $60,000	24	12.9%
	Between $60,001 and $75,000	14	7.5%
	$75,000 or more	36	19.4%
Precarious Housing Situation			
	Yes	19	9.8%
	No	174	90.2%

Number reflects the actual number of respondents who answered the question. Percent may not equal 100% due to rounding.

**Table 2:** Descriptive statistics for variable by total sample and by trimester subsamples.

		Total, n = 195	First Trimester, n = 14	Second Trimester, n = 54	Third Trimester, n = 127
		N (%)	N (%)	N (%)	N (%)
**Routine Weather Checking**					
	Daily	76 (39.0%)	5 (35.7%)	18 (33.3%)	53 (41.7%)
	Most Days	62 (31.8%)	5 (35.7%)	21 (38.9%)	36 (28.3%)
	Sometimes	46 (23.6%)	3 (21.4%)	12 (22.2%)	31 (24.4%)
	Rarely/Never	11 (5.6%)	1 (7.1%)	3 (5.6%)	7 (5.5%)
**Event-Driven Weather Information Seeking**					
During Pregnancy					
	Needs More	87 (44.6%)	6 (42.9%)	24 (44.4%)	57 (44.9%)
	Does Not Need More	108 (55.4%)	8 (57.1%)	30 (55.6%)	70 (55.1%)
During Extreme Heat					
	Needs More	100 (51.3%)	5 (35.7%)	30 (55.6%)	65 (51.2%)
	Does Not Need More	95 (48.7%)	9 (64.3%)	24 (44.4%)	62 (48.8%)
**Physiological Heat-Health Protective Actions**					
Limit Time in the Sun					
	More Than Normal	158 (81.0%)	12 (85.7%)	38 (70.4%)	108 (85.0%)
	Not More Than Normal	37 (19.0%)	2 (14.3%)	16 (29.6%)	19 (15.0%)
Hydration					
	More Than Normal	163 (83.6%)	11 (78.6%)	43 (79.6%)	109 (85.8%)
	Not More Than Normal	32 (16.4%)	3 (21.4%)	11 (20.4%)	18 (14.2%)
Spend Time in Air Conditioning					
	More Than Normal	153 (78.5%)	12 (85.7%)	38 (70.4%)	103 (81.1%)
	Not More Than Normal	42 (21.5%)	2 (14.3%)	16 (29.6%)	24 (18.9%)
**Exposure Adaptation by Changing Plans**					
	Always	53 (27.2%)	6 (42.9%)	14 (25.9%)	33 (26.0%)
	Often	68 (34.9%)	2 (14.3%)	23 (42.6%)	43 (33.9%)
	Sometimes	59 (30.3%)	4 (28.6%)	14 (25.9%)	41 (32.3%)
	Rarely/Never	15 (7.7%)	2 (14.3%)	3 (5.6%)	10 (7.9%)

Notes: n means sample size, N means Number, % means Percent. Percents may not equal 100.0% due to rounding.

**Table 3:** Bivariate analyses between routine weather information seeking and event-driven weather information seeking by trimester.

	Routine Weather Information Seeking							
Event-Driven	Total		First Trimester		Second Trimester		Third Trimester	
	$\chi^{2}$	D	$\chi^{2}$	D	$\chi^{2}$	D	$\chi^{2}$	D
During Pregnancy	3.21	−0.09	1.48	−0.24	3.36	−0.12	0.86	−0.06
During Extreme Heat	5.88	−0.18 *	3.55	−0.22	0.66	−0.02	8.89 *	−0.12 *

Note: * *p* < 0.05.

**Table 4:** Bivariate analyses between weather information seeking forms and limiting time in the sun by trimester.

Weather Information Seeking	Limiting Time in the Sun							
	Total		First Trimester		Second Trimester		Third Trimester	
	$\chi^{2}$	D	$\chi^{2}$	D	$\chi^{2}$	D	$\chi^{2}$	D
Routine	6.04	−0.08	7.47	−0.21	3.62	−0.15	1.33	−0.03
During Pregnancy	5.72 *	0.14 *	1.75	0.25	3.48	0.23 *	1.60	0.08
During Extreme Heat	16.08 ***	0.23 ***	1.30	0.22	8.60 **	0.37 **	8.12 **	0.18 **

Note: * *p* < 0.05, ** *p* < 0.01, *** *p* < 0.001.

**Table 5:** Bivariate analyses between weather information seeking and staying hydrated by trimester.

	Staying Hydrated							
Weather Information Seeking	Total		First Trimester		Second Trimester		Third Trimester	
	$\chi^{2}$	D	$\chi^{2}$	D	$\chi^{2}$	D	$\chi^{2}$	D
Routine	3.91	−0.05	5.29	−0.28	2.19	−0.10	8.65 *	−0.01
During Pregnancy	4.21 *	0.11 *	0.14	0.08	3.86 *	0.22 *	1.13	0.07
During Extreme Heat	4.38 *	0.11 *	2.12	0.33 *	4.48 *	0.23 *	0.38	0.04

Note: * *p* < 0.05.

**Table 6:** Bivariate analyses between weather information seeking and spending time in air conditioning by trimester.

Weather Information Seeking	Spending Time in Air Conditioning							
	Total		First Trimester		Second Trimester		Third Trimester	
	$\chi^{2}$	D	$\chi^{2}$	D	$\chi^{2}$	D	$\chi^{2}$	D
Routine	5.79	−0.10 *	7.47	−0.21	1.22	−0.07	4.02	−0.10
During Pregnancy	5.57 *	0.20 *	1.75	0.25	1.60	0.16	2.96	0.12
During Extreme Heat	13.49 ***	0.32 ***	1.30	0.22	8.60 **	0.12 **	5.74 *	0.27 *

Note: * *p* < 0.05, ** *p* < 0.01, *** *p* < 0.001.

**Table 7:** Bivariate analyses between weather information seeking, immediate physiological protective actions, and exposure adaptation of changing plans by trimester.

	Changing Plans							
	Total		First Trimester		Second Trimester		Third Trimester	
	$\chi^{2}$	D	$\chi^{2}$	D	$\chi^{2}$	D	$\chi^{2}$	D
**Weather Information Seeking**								
Routine	15.34	−0.19 *	6.53	−0.09	11.98	−0.24 *	11.94	−0.19 *
During Pregnancy	12.35 ***	0.18 *	1.75	0.25	3.75	0.15	7.26	0.19
During Extreme Heat	12.84 ***	0.27 ***	4.93	0.58 *	1.22	0.15	9.57 *	0.29 **
**Immediate Physiological Protective Actions**								
Limit Sun	10.34 *	0.28 **	1.07	−0.08	0.73	0.13	18.16 ***	0.50 ***
Stay Hydrated	11.21 *	0.22	5.09	0.73 *	2.30	−0.11	12.91 **	0.32 *
Air Conditioning	3.10	0.17	1.10	−0.08	2.14	0.19	4.14	0.20

Note: * *p* < 0.05, ** *p* < 0.01, *** *p* < 0.001.

## Data Availability

Data and documentation used in this study will be made available in the *DesignSafe* repository (https://www.designsafe-ci.org/ (accessed on 8 June 2026)) upon publication.

## Abbreviations

The following abbreviations are used in this manuscript:

C	Celsius
F	Fahrenheit
NOAA	National Oceanic and Atmospheric Administration
PADM	Protective Action Decision Model
